# How Is Bone Regeneration Influenced by Polymer Membranes? Insight into the Histological and Radiological Point of View in the Literature

**DOI:** 10.3390/membranes14090193

**Published:** 2024-09-11

**Authors:** Alexandra Papuc, Simion Bran, Marioara Moldovan, Ondine Lucaciu, Gabriel Armencea, Grigore Baciut, Cristian Dinu, Florin Onișor, Winfried Kretschmer, Mihaela Baciut

**Affiliations:** 1 Department of Maxillofacial Surgery and Implantology, Iuliu Hațieganu University of Medicine and Pharmacy, Iuliu Hossu Str. 37, 400029 Cluj-Napoca, Romania; 2 Raluca Ripan Institute for Research in Chemistry, Fantanele 30, Babeș Bolyai University, 400294 Cluj-Napoca, Romania; 3 Department of Oral Health, Iuliu Hațieganu University of Medicine and Pharmacy, Victor Babes Str. 15, 400012 Cluj-Napoca, Romania; 4 Klinik fur Mund-, Kiefer- und Plastische Gesichtschirurgie, Alb Fils Kliniken GmbH, Goppingen, Baden-Wurttemberg, 73035 Göppingen, Germany

**Keywords:** polymeric scaffolds, critical bone defect, bone regeneration, mechanical properties

## Abstract

The aim of this study was to analyze published works that investigate the in vivo bone regeneration capacity of polymeric membranes loaded with active substances and growth factors. This scoping review’s purpose was to highlight the histological and radiological interpretation of the locally produced effects of the polymer membranes studied so far. For the selection of the articles, a search was made in the PubMed and ScienceDirect databases, according to the PRISMA algorithm, for research/clinical trial type studies. The search strategy was represented by the formula “((biodegradable scaffolds AND critical bone defect) OR (polymers AND mechanical properties) OR (3Dmaterials AND cytotoxicity) AND bone tissue regeneration)” for the PubMed database and “((biodegradable scaffolds AND polymers) OR (polymers AND critical bone defects) OR (biodegradable scaffolds AND mechanical properties) AND bone tissue regeneration)” for the ScienceDirect database. Ethical approval was not required. Eligibility criteria included eight clinical studies published between 2018 and 2023. Our analysis showed that polymer membranes that met most histopathological criteria also produced the most remarkable results observed radiologically. The top effective scaffolds were those containing active macromolecules released conditionally and staged. The PLGA and polycaprolactone scaffolds were found in this category; they granted a marked increase in bone density and improvement of osteoinduction. But, regardless of the membrane composition, all membranes implanted in created bone defects induced an inflammatory response in the first phase.

## 1. Introduction

Recently, the elderly population has increased, with an implicit increase in bone disease prevalence. In 2010, globally, approximately 524 million people were 65 years old or older, representing 8% of the world’s population. At this rate, by 2050 this figure is expected to triple to 22% [1]. Fractures or loss of bone tissue, which cause limited mobility and severe disability, will represent one of the major public health problems. In the current social context, one of the most promising strategies for the treatment of bone lesions is regenerative medicine [2]. Bone grafts represent the second most common tissue transplanted in the United States [3]. The gold standard treatment in these situations is autograft tissue harvested from the patient, most commonly from the iliac crest, from the proximal area of the tibia or the distal femur [4]. Even though they present all these advantages and are considered the gold standard in bone regeneration, autografts and allografts can be accompanied by infections, bleeding and scars; for this reason, the latest research has focused on the development and optimization of matrices/scaffolds that mimic the cellular matrix support. Bone regeneration is a complex process dependent on molecular involvement, which promotes the migration, proliferation and differentiation of mesenchymal stem cells. For this reason, several biological products, characterized by a porous network, capable of inducing and stimulating bone cells have been developed over the years [5,6]. Adapting the surface of biomaterial scaffolds has been a key strategy to modulate cellular involvement that supports the tissue healing process. In particular, nano-topological surfaces have been shown to regulate various stem cell behaviors such as initial adhesion, spreading and lineage specification [5,6,7]. Among the biological materials most used in the last decade are polymeric membranes, due to the ease with which the size of the pores can be controlled at the time of their formation—a design that is recommended in applications involving tissue engineering because of their properties similar to the replaced tissue [8,9,10]. 

Many advantages of these materials stand out, such as the wide availability on the market, resistance to external forces, ease of clinical manipulation in minimally invasive procedures, minimal resorption rate and promising aesthetic results due to the possibility of prefabrication. At the same time, one of the most important drawbacks of polymeric scaffolds is given by the presence of residual monomers released post-procedurally. It is known that residual monomers can induce allergic reactions and tissue cytotoxicity [11]. The most accepted definition was given by William in 1987 as follows: “a non-viable material used in a biomedical device intended to interact with biological systems” [12,13,14,15,16,17]. In this field, the most common scaffolding approaches aim at the in vitro support of cells on the one hand and tissue stimulation on the other by adding active factors such as drugs, hemostatic or growth factors [18,19,20,21]. It is becoming increasingly vital to use composite polymer membranes in regeneration. With the dimensional stability given by modified structures, the composite scaffolds contribute to the regeneration processes by releasing the active molecules. The addition of growth factors and active biomolecules came from the desire to improve the properties of resorbable membranes, which often have several disadvantages such as rapid degradation rate and poor volume stability, along with rapid fragmentation and degradation after gingival dehiscence with membrane exposure and associated low bone regeneration. Traditional fabrication methods such as fiber bonding, emulsification, particulate/salt leaching, separation/inversion, freeze drying, etc., are accompanied by similar various disadvantages like the poor control of the pore size, shape and architecture, causing a poor relationship between the membrane and the tissue. To overcome those disadvantages, computer technology has been used to design and fabricate scaffolds with controlled properties [20,21]. A wide variety of scaffolds have been developed according to the main requirements of biocompatibility, osteoinduction and osteoconduction. 

Moreover, in order to achieve effective tissue regeneration, a membrane should guarantee dimensional stability and should be biodegradable, easy to manufacture and process. Also, the presence of pores larger than 300 µm is necessary to obtain adequate vascularization, which optimizes the bone regeneration process [2,22]. So far, it has been shown that the initial interaction occurs between polymers and mesenchymal cells (granulocytes, monocytes and lymphocytes). These cells use fibronectin to anchor collagen in the extracellular matrix. Recently, tissue transglutaminases (tTG)—enzymes known for their increased affinity for fibronectin—have been used to obtain a biocompatible polymer response [23]. Thus, through the response of integrins in the extracellular matrix, a local biological response will be initiated. On the other hand, it has been demonstrated that proto-oncogenes such as (c- fos, c-jun) will simultaneously activate osteoblasts as a mechanical response to local stimuli. Due to these local biological effects (intensively studied in the last decade), polymers are mainly used for fracture immobilization, bone regeneration, ligament fixation, release of active substances or cartilage restructuring. 

There are two main types of polymers: natural and synthetic [4,24]. The main natural polymers involved are proteins (collagen, fibrin) and polysaccharides (chitosan, alginate, hyaluronic acid or cellulose). They contain biofunctional macromolecules that guarantee cellular bioactivity and tissue remodeling. On the other hand, the main disadvantages would be immunogenic response, the risk of microbial contamination (through the release of toxins), uncontrollable degradation rate and mechanical resistance, which limit their application on large surfaces [2,25,26]. Synthetic polymers are structurally characterized according to composition. These advantages provide predictability and reproducibility required by specific applications as seen in Table 1. However, compared with natural polymers, they have reduced bioactivity and osteoconductivity. Among the most widely used synthetic polymers are the aliphatic polyesters poly(ε-caprolactone) (PCL), polylactide (PDLA, PLLA) and poly(lactide-co-glycolide) (PLGA) [12,23]. The purpose of the current scoping review is to analyze published works that investigate the in vivo bone regeneration capacity of polymeric scaffolding. Even if, over time, there have been numerous publications on this subject, this study will focus on polymeric membranes loaded with active substances and growth factors. In addition, this analysis aims to present the most relevant bone changes observed following the use of membranes, as along with highlighting the histological and radiological interpretation of the locally produced effects of the polymer membranes studied so far.

## 2. Materials and Methods

For the selection of the articles, two researchers independently searched PubMed and ScienceDirect databases, according to the PRISMA algorithm, for research/clinical trial type studies. The search strategy was represented by the formula “((biodegradable scaffolds AND critical bone defect) OR (polymers AND mechanical properties) OR (3Dmaterials AND cytotoxicity) AND bone tissue regeneration)” for the PubMed.gov database and “((biodegradable scaffolds AND polymers) OR (polymers AND critical bone defects) OR (biodegradable scaffolds AND mechanical properties AND bone tissue regeneration)” for the ScienceDirect database. Different strategies were used for these databases in order to cover a wider field of the chosen theme. Also, the use of different strategies justifies why there were 0 duplicate articles. Ethical approval was not required.

Subsequently, two researchers conducted the search and independently filtered studies based on titles and abstracts using the inclusion and exclusion criteria described above, and then they screened articles by reviewing the full text of the studies that had previously been filtered during the first stage. If no agreement could be reached, a third senior author designated whether the article should be included or excluded. In the first instance, 699 articles were selected from the 2 mentioned databases in accordance with the search terms previously mentioned. After the initial search based on titles, 566 articles were excluded. The abstracts were evaluated for 143 articles and a filter was applied regarding the time period in which the articles were published, choosing only publications between 2018 and 2023. The original articles were defined as clinical studies carried out in the laboratory or on animal specimens. Out of 143 articles, 113 were analyzed and eliminated not meeting all the inclusion criteria, and 22 reviews and meta-analyses were excluded from this study because they were not in accordance with the proposed research. In the end, only 8 articles that met the eligibility criteria were included in this scoping review.

In the next stage, the eligibility criteria were applied, which defined the final number of articles used in this study.

(1) Articles were available in full text format;
(2) Articles written in English;
(3) First author of the original document;
(4) Research/clinical trial-type studies;
(5) Comparison of two or more membranes;
(6) Natural or synthetic polymers used as replacement membranes for regenerative purposes;
(7) Results presented as quantitative data;

Articles that did not meet the criteria were excluded from the full text analysis.

(1) Reviews, meta-analysis, cohort studies, case series, descriptive studies, opinion articles and abstracts;
(2) Duplicated articles;
(3) Studies involving only one scaffold;
(4) Articles that incorporated various inconclusive data.

At the end of full test screening, the two reviewers exchanged notes and compared their selections in order to unify the screening criteria.

Qualitative evaluation

(1) In order to minimize the risk of bias, all of the included research papers were subjected to a qualitative assessment using a diagnostic accuracy research quality assessment tool (QUADAS-2). Every article included was thoroughly analyzed by two independent examinators, and every domain was rated either “high”, “unclear” or “low” according to their bias risk.

## 3. Results

A total of 699 articles (641 on PubMed.gov and 58 on ScienceDirect) were retrieved from databases. There were no duplicated articles. In December 2023, two authors independently extracted the general characteristics of the included studies, which were then recorded on a predefined list. The extracted data included the following: membrane types, biodegradable materials, animal subjects, histological and imaging analyses, follow-up time, bone regeneration data, complications and the first author of the original documents.

**Figure 1:**
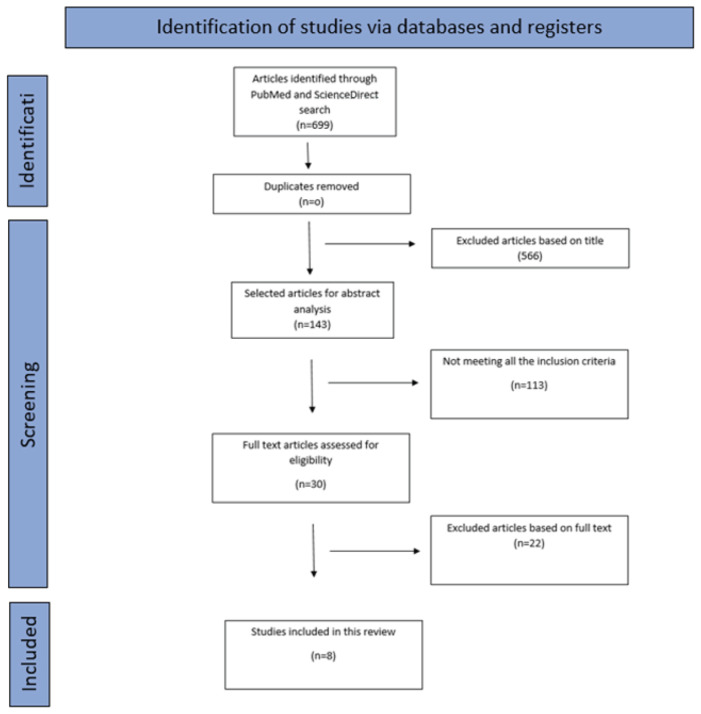
PRISMA flow-chart diagram for the selection of the 8 articles included in the review.

One hundred and forty-three articles were selected for abstract analysis; the rest were excluded after title examination. Due to not meeting all the aforementioned criteria, 113 publications were excluded from the current study. As a result, the remaining 8 articles were assessed for eligibility and selected based on full text (Figure 1).

All studies aimed to implement new polymeric membranes for restoration of bone defects created in an animal specimen. Studies have followed the evolution of bone regeneration induced by various polymeric materials. Selection criteria focused not only on the histopathological response but also on the radiologically evaluated bone density. In order to be able to characterize the ideal membrane, the present work proposed the comparison of the eight studies, according to the above-mentioned parameters.

In 2020, B. Bakhtiarimoghadam [38] investigated the effect of cultured collagen, enhanced with or without mesenchymal cells or platelet-rich plasma, on the regeneration of full-thickness in a rabbit animal model. One hundred and sixty-one rabbits were used in this study and randomly divided into sixteen groups of ten rabbits, including treatment-free defect, PRP, MSC, CH, HA/Co, In/HA/Co, CH + PRP, HA/Co + PRP, In/HA/Co + PRP, CH + MSC, HA/Co + MSC, In/HA/Co + MSC, CH + PRP + MSC, HA/Co + P + MSC and In/HA/Co + MSC + PRP. A 5 cm craniomedial incision was made at the level of the right limb. After the muscle dissection, a bone defect with a diameter of 5 mm was made and the chosen material was positioned. For an imaging evaluation, post-interventional X-rays were performed and the results were evaluated using the modified Lane and Sandhu scale. Also, the sections were blindly evaluated and scored by an expert according to the Han system. He concluded that, although plaque-like HA particles are homogenously distributed in situ, HA/CO scaffold compared with HA/CO scaffold mixed with powder and had a composition similar to bone, and the structure created by addition of collagen is unstable over time due to the high speed of resorption.

D. Lee, in 2021, [39] demonstrated the synergic effects of two bioactive molecules—morphogenetic protein 2 (BMP-2) and alendronate (ALN)—through their sequential release at the level of the implantation site. Forty-two rats were used, and an 8 mm defect in the calvary bone was made. The animals were randomly divided into seven groups. To evaluate new bone formation, images were obtained using micro-computerized tomography. Imagistics was performed on the scanner at an isotropic size of 9 pm voxel with an X-ray tube. The results of the study showed that the use of two bioactive substances led to an improvement in bone functionality and safety but also to an acceleration of bone matrix formation.

In 2019, a study led by A. Tateno [40] evaluated the regeneration potential in critical size mandibular defect of a type I collagen scaffold made from a recombinant peptide with Arg-Gly-Asp. Fifteen male rats were used for the in vivo experiment. An incision was made in the lower vestibule, and a bone defect with the size of 4 × 3 × 1 was made distal to the incisor. RCP-block scaffolds of appropriate size were placed in the defects and covered with a membrane. The images were obtained from individual rats immediately after surgery and every week thereafter, up to 8 weeks after surgery. The images were reconstructed in 3D images with i-View. Bone volume was measured in regions of interest (ROI) in voxel images using the bone volume measurement software package. The findings suggested the DFAT is effective for bone formation, but more studies are needed to confirm that they are a promising source for bone regeneration.

G.E. Vigni [41], used nine rabbits to determine the bone regeneration potential by placing a polybutylene succinate-based membrane at the level of the frontal bone defects. Two 8 mm circular defects were made and, at the end of the procedure, each animal had two defects: one control left to heal spontaneously and the other covered by the membrane. Both histological examination and CT scans confirmed the presence of osteoblasts and mineralized tissue in the scaffold-treated defect.

In March 2018, Y. He [42] created a membrane based on collagen due to its biocompatibility and osteoconductivity by using active biomolecules as alendronate or bone marrow stem cells to improve bone osteoinduction and mineralization. Defects in the calvary bone were created and scaffolds were placed to stimulate osteoregeneration. The study indicated that bone marrow stem cells were able to attach and participate in cellular differentiation processes independently of external factors. In all cases, the bone density obtained was superior in the case of membrane defects compared with the blank defects.

In 2021, due to the deficient osteoinduction and the hydrophobic surface of PLGA membranes, C. Fu [43] created a scaffold to improve the bone repair ability of PLGA by function modification by blending L-lysine functionalized graphene oxide. New Zealand white rabbits were used. The 20 mm bone defects were made in the radius and the scaffold material was trimmed and the defect site implanted. The study demonstrated that simultaneously using more bioactive molecules is a promising strategy in bone regeneration.

K-H H., in 2020 [44], developed a biodegradable calcium silicate/calcium sulfate/polycaprolactone scaffold. Additionally, he loaded the membranes with BMP-2 through a one-step immersion procedure. The results indicated a slow degradation over time of the scaffold with the gradual release of active substances, a fact that led to a good stability over time promoting the formation of new blood vessels and bone regeneration.

In 2020, a polycaprolactone membrane was created with 3D technology by S. Lee [45], containing heterogeneous pores for more effective bone ingrowth. The biomaterial was subsequently placed at the mandibular level. The results showed increased rigidity and fixation to the residual bone due to the 3D structure. Loose porous areas in BMP-2 (recombinant human bone morphogenetic protein-2) and ABP (autogenous bone particles) groups were fixed by connective tissue.

Following the selection of the articles, the bone regeneration process was followed at different time intervals from the radiological and histopathological point of view. 

After synthesizing the data on the cellular effects produced by the polymer membranes used, the same process was applied to summarize the information regarding the radiological changes that occurred.

## 4. Discussion

Over the past two decades, researchers have investigated the use of various scaffolds and biomaterials to facilitate drug delivery and bone tissue engineering using scaffolds and biomaterials. Composite scaffolds, nano-biomaterials and platelet-rich autologous fibrin have been identified as effective drug delivery systems that can accelerate tissue regeneration and healing by promoting osteogenesis. These osteogenic mechanisms associated with drug delivery may improve the efficacy of therapeutic applications.

As described in the studies, rats and rabbits are the most common animal models used. This is because rat models are extraordinarily useful for conducting basic skeletal research and are reliable and cost-effective alternatives to dogs and non-human primates. In combination with the directed differentiation of MSCs in damaged tissue, this paracrine property is essential for tissue repair and regeneration. Also, the use of rabbits is justified by low costs, their increased resistance and, last but not least, by the similarity between their bone constitution and children’s bone constitution.

The purpose of this study was to investigate the potential ability of polymeric membranes through the in situ mineralization of the bioactive particles in their structure and to compare the histopathological effects induced at the implantation site, along with radiologically interpreting the bone changes.

All animals were housed in individual cages and fed according to a standard diet imposed by the laboratory. They were allowed the freedom to move in cages at will. Also, in B. Bakhtiarimoghadam’s study, the animals were treated with anti-parasitic drugs for 1 week before surgery, while animals in the other seven studies were not given pre-surgical medication.

1. Histopathologic response

Despite the remarkable development of imaging and computational technologies, the histopathological examination remains a standard evaluation technique for cell growth and estimation of a pathological process.

In this study a total of eight papers were compared based on the histopathologic response in order to provide evidence based on cell morphology and cell proliferation in the newly formed bone cavities. All the samples were washed, fixed and sectioned, then stained as follows: in seven studies, tissue containing scaffolds was removed en bloc and fixed in H&E (hematoxylin and eosin staining) and a modified Masson’s trichrome stain kit, respectively. Trichrome staining in blue was used to identify collagen. In addition, one of the studies used sample groups treated with formaldehyde. The scaffolds were then examined at 2–4, 8–12 and 24 weeks, according to the processing protocol. Regardless of the membrane composition, for the first interval, all the scaffold-treated defects were filled with fibrous tissue and showed inflammatory cells along the periphery. ALN and A + BMP2 groups showed more inflammatory cells, along with fibroblast-like cells in the defect area, preventing new bone formation. Notable were the modified collagen membranes with alendronate sodium, which provided a mature bone structure in the center of the bone defect from an early stage. Between 8 and 12 weeks, mostly pure collagen modified membranes were most affected, and the scaffold-treated defect was filled with fibrous tissue and lamellar bone without cartilage, lacking typical bone formation. All other membranes analyzed in this work produced notable results. The phosphorylated scaffolds proved to have excellent osteoinduction properties and the PLGA group presented lamellar bone, new bone tissue in the defect and more mature collagen fibers. The only membrane that was followed for 24 weeks was a biodegradable membrane made of succinate polybutylene. Here, in addition to the areas of osteonecrosis present at the periphery, immature bone cells and osteoblasts involved in osteosynthesis processes were observed (Table 2).

Polymer membranes have the capability to facilitate cell attachment, proliferation, new bone growth and biodegradation of the scaffold. Moreover, the inclusion of biofunctional macromolecules into the polymer matrix creates a suitable blend of biological, chemical and physical proprieties in the biomaterial that closely mimics the bone’s natural ECM. These hybrid membranes, with optimal structures for hosting additives and generating local immune responses, suitable mechanical proprieties, biocompatibility and controlled elasticity, prove that the manufacture and preparation of materials used is an essential stage in the healing process.

2. Radiological response

Radiography remains the fastest and easiest way to evaluate bone lesions, but also to evaluate bone density or bone changes. In this part of this study, we focused on tracking the bone changes that occurred as a result of scaffolds used as described in the eight debated articles. To evaluate bone regeneration and remodeling of the created defect, we assessed the accumulation and maturation of bone-like tissues in the bone defect at 2–4, 8–12 and 24 weeks after membrane implantation, using micro-CT imaging data from the eight articles (Table 3).

In the first reference interval, the ALN-BMP2 group stood out for the formation of new bone tissue, owing to alendronate sequential release. After more time since implantation, the most significant results were obtained using PCL and PLGA membranes, where lamellar bone formation was excellent, osteoinduction was present and bone density improved, close to normal bone density. On the other hand, in the case of collagen membranes, scaffold stability and bone stimulation were rendered by additives such as alendronate and peptide, which stabilized the membrane and implicitly led to bone formation and significant radiological union. 

Also, all studies indicated that the bone volume did not significantly increase until 8 weeks. Beginning with week 8, the potential for regeneration in bone critical size defects was easily identified histologically and with micro-CT.

In all cases, micro-CT showed that scaffolds containing active biomolecules improved tissue repair and cell ingrowth in pores in order to fill the defect region. 

The limitation of this study in the aspect of gathering data was due to a large number and variability in study designs of articles regarding this topic. This led to choosing only publications between 2018 and 2023. Also, the use of various imaging techniques and protocols was incompatible with the starting idea of comparing the results in a systematic way. This feature is not a limitation per se, but rather recognition that no simple method works well and that this issue must be addressed in a narrative-based manner.

## 5. Conclusions

There is continuous dynamic development in tissue engineering towards new scientific trends for improved regeneration. Innovations in manufacturing processes enable the production of membranes with good stability, controlled biodegradability, elasticity and strength over time.

Our analysis showed that polymer membranes that met most histopathological criteria also produced the most remarkable results when radiologically observed. The top effective scaffolds were those containing active macromolecules released conditionally and staged. The PLGA and polycaprolactone scaffolds were found in this category and they granted a marked increase in bone density and improvement in osteoinduction.

Polymer membranes have the capability to facilitate cell attachment, proliferation, new bone growth and biodegradation of the scaffold. Moreover, the inclusion of biofunctional macromolecules into the polymer matrix creates a suitable blend of biological, chemical and physical proprieties in the biomaterial that closely mimics the bone’s natural ECM. These hybrid membranes, with optimal structure for hosting additives and generating local immune responses, suitable mechanical proprieties, biocompatibility and controlled elasticity, prove that the manufacture and preparation of materials used is an essential stage in the healing process.

Regardless of the membrane composition, all membranes implanted in created bone defects induce in the first phase an inflammatory response resulting in the appearance of cell chains on the periphery of defects and a tissue rich in fibrin. However, the most suitable membranes to produce osteoinduction and stimulate local bone regeneration are membranes that incorporate conditionally released biomolecules. These additives stimulate osteoconduction and cell proliferation over a longer period of time, ensuring membrane stability and controlled degeneration.

## Figures and Tables

**Table 1:** The main advantages and disadvantages of polymeric membranes.

Material	Advantages	Disadvantages	Reference
**Natural polymers**			
**Collagen** **(CO)**	Biocompatible Enzymatically biodegradable Possibility of injection FDA approved	Low mechanical resistance Difficult to disinfect Difficult to handle Increased risk of inflammation Rapidly biodegradable	[4,25,27]
**Gelatin**	Biocompatible Biodegradable Osteoconductive	Low mechanical resistance Reduced stability	[28,29]
**Silk fibrin**	Increase mechanical resistance Thermal stability Non-toxic Biocompatibility	Limited biological adhesion	[4,28,30]
**Chitosan** **(CTS)**	Biocompatible Porous structure Antibacterial Non-toxic Osteoconductive	Low mechanical resistance Low stability	[4,27,28]
**Alginate**	Biocompatible Easy to handle Controlled release of active substances	Limited biological adhesion	[4]
**Hyaluronic acid**	Biocompatible Biodegradable Increased viscosity Easy to handle	Low mechanical resistance	[2,4,31,32]
**Cellulose**	Biocompatible Thermal stability	Unstable structure	[4,33]
**Synthetic polymers**			
**PCL**	Biocompatibility Superior mechanical strength Low degradation rate	Hydrophobic Low bioactivity	[4,34]
**PLA**	Cytocompatibility Thermal stability Biodegradable	Reduced mechanical strength compared with PCL	[4,35]
**PLGA**	Controlled degradation Biodegradable Biocompatible	Reduced osteoconductivity	[4,36,37]

**Table 2:** Histopathological response of various polymeric membranes used.

Author/Publication Year	Materials	2–4 Weeks	8–12 Weeks	24 Weeks
**1.** Behnam Bakhtiarimoghadam/2020	**Untreated defect**	No newly bone formed.	Significantly lower score than the rest of the groups.	No data
	**Type I collagen scaffolds**	Newly formed cartilage and fibrous tissue.	Fibrous tissue, lamellar bone without cartilage.	
**2.** Dongtak Lee/2021	**Alendronate treated defect**	Fibrous tissue, without much osseous tissue.		No data
	**Alendronate and BMP-2 encapsulated**	Inflammatory cells prevented the new bone formation.		
	**BMP-2 and alendronate encapsulated**	Mature bone with lamellar structure.		
**3.** Atsushi Tateno/2019	**Type I collagen peptide**	No data	New bone tissue in all samples. In the DFAT/RCP group, significantly more bone was regenerated than in the ASC/RCP group.	No data
**4.** Giulio Edoardo Vigni/2022	**Untreated defect**		Rich in fibrous tissue, bone tissue partially infiltrated at the periphery.	Osteonecrosis visible, poorly mineralized and fragmented trabeculae
	**Polybutylene succinate (PBS)**		Rich in new bone tissue, fibrous tissue undergoing mineralization.	Osteonecrosis present. Osteosynthesis with immature bone and osteoblasts.
**5.** Yingcong He/2018	**Untreated defect:**	Fibrous tissue was detected.	Lack of typical bone formation.	No data
	**Type I collagen with alendronate**	Mature bone structure found in the central bone defect.	Excellent osteoinduction properties.	
**6.** Chuan Fu/2021	**Untreated defect**	No data	Fibrous tissue, hyperplasic connective tissue.	No data
	**PLGA**		Lamellar bone, new bone tissue in bone defect. More mature collagen fibers.	
**7.** Kuo-Hao Huang/2021	**Polycaprolactone**	No data	MS/CS/B increased bone tissue, stimulated collagen formation and osteoid tissue.	No data
**8.** Sanghoon Lee/2020	**Polycaprolactone/** **beta tricalcium phosphate**	No data	Loose porous area in BMP-2 (recombinant human bone morphogenetic protein-2) and ABP (autogenous bone particles) groups had been fixed by connective tissue.	No data

**Table 3:** Radiological response of various polymeric membranes used.

Nr.	Materials	2–4 Weeks	8–12 Weeks	24 Weeks
**1.** Behnam Bakhtiarimoghadam/2020	**Type I collagen scaffolds**	No data	Bone formation and significantly radiological union.	No data
**2.** Dongtak Lee/2021	**Alendronate treated defect**	*p* < 0.01	*p* < 0.001	No data
	**Alendronate and BMP-2 encapsulated**	Bone volume fraction was the highest in the BMP-2 and alendronate encapsulated group because of the sequentially release of ALN.		
**3.** Atsushi Tateno/2019	**Type I Collagen peptide**	No data	Significant amount of new bone formation in the DFAT (lipid free differentiated fat)/RCP GROUP compared with ASC (adipose-derived stem cells)/RCP. *p* < 0.001	No data
**4.** Giulio Edoardo Vigni/2022	**Polybutylene succinate (PBS)**	Little appreciable healing in all defects.	No statistically significant results due to a limited number of samples.	
**5.** Yingcong He/2018	**Untreated defect**	New bone tissue formation in scaffolds.		No data
	**Type I collagen with alendronate**	The new bone tissue formation was higher in the Col/Aln group than in Col/control groups. Micro-CT showed that the phosphorylated scaffolds were filled with more bone than the unmodified collagen scaffolds.		
**6.** Chuan Fu/2021	**Untreated defect group**	No data	Bone tissues was visible in the center of the bone defect.	No data
	**PLGA**		Bone tissue was more significant, the radius was close to that of normal bone tissue.	
**7.** Kuo-Hao Huang/2021	**Polycaprolactone**	No data	Bone tissue formation, excellent osteoinduction, improved bone density in MS/CS/B groups compared with the MS/CS scaffolds.	No data
**8.** Sanghoon Lee/2020	**Polycaprolactone/** **beta tricalcium phosphate**	No data	All groups presented new bone tissue formation. Significantly, the highest bone formation occurred in the group loaded with rhBMP-2 (recombinant human bone morphogenetic protein-2). *p* < 0.05.	No data

## Data Availability

The original contributions presented in the study are included in the article, further inquiries can be directed to the corresponding author.

## References

[1] Padilla Colón C.J., Molina-Vicenty I.L., Frontera-Rodríguez M., García-Ferré A., Rivera B.P., Cintrón-Vélez G., Frontera-Rodríguez S. (2018). Muscle and Bone Mass Loss in the Elderly Population: Advances in diagnosis and treatment. J. Biomed..

[2] Donnaloja F., Jacchetti E., Soncini M., Raimondi M.T. (2020). Natural and synthetic polymers for bone scaffolds optimization. Polymers.

[3] Baldwin P., Li D.J., Auston D.A., Mir H.S., Yoon R., Koval K.J. (2019). Autograft, Allograft, and Bone Graft Substitutes: Clinical Evidence and Indications for Use in the Setting of Orthopaedic Trauma Surgery. J. Orthop. Trauma.

[4] Bharadwaz A., Jayasuriya A.C. (2020). Recent trends in Application of widley used natural and syntetic polymer nanocomposites in bone tissue regeneration. Mater. Sci. Eng. C.

[5] Malik R., Garg T., Goyal A.K., Rath G. (2016). Diacerein-loaded novel gastroretentive nanofiber system using PLLA: Development and in vitro characterization. Artif. Cells Nanomed. Biotechnol..

[6] Gurtner G.C. (2018). Plastic Surgery, Volume 1: Principles.

[7] Patel K.D., Kim T.H., Mandakhbayar N., Singh R.K., Jang J.H., Lee J.H., Kim H.W. (2020). Coating biopolymer nanofibers with carbon nanotubes accelerates tissue healing and bone regeneration through orchestrated cell- and tissue-regulatory responses. Acta Biomater..

[8] Moldovan M., Campian R. (2021). Electrospun Membranes Based on Polycaprolactone. Materials.

[9] Loison-Robert L.S., Tassin M., Bonte E., Berbar T., Isaac J., Berdal A., Simon S., Fournier B.P.J. (2018). In vitro effects of two silicate-based materials, Biodentine and BioRoot RCS, on dental pulp stem cells in models of reactionary and reparative dentinogenesis. PLoS ONE.

[10] Karacan I., Ben-Nissan B., Wang H.A., Juritza A., Swain M., Mueller W., Chu J., Stamboulis A.M.I., Taraschi V. (2019). Mechanical testing of antimicrobial biocomposite coating on metallic medical implants as drug delivery system. Mater. Sci. Eng. C.

[11] Miron A.E., Moldovan M., Prejmerean C.A., Prodan D., Vlassa M., Filip M., Badea M.E., Moldovan M.A. (2020). New Antimicrobial Biomaterials for the Reconstruction of Craniofacial Bone Defects. Coatings.

[12] Perez-Puyana V., Jiménez-Rosado M., Romero A., Guerrero A. (2020). Polymer-based scaffolds for soft-tissue engineering. Polymers.

[13] Politi S., Carotenuto F., Rinaldi A., Di Nardo P., Manzari V., Albertini M.C., Araneo R., Ramakrishna S., Teodori L.S. (2020). ECM-Based Electrospun Biomaterials for Skeletal Muscle Regeneration. Nanomaterials.

[14] Opris H., Dinu C., Baciut M., Baciut G., Mitre I., Crisan B., Armencea G., Prodan D.A., Bran S. (2020). The influence of eggshell on bone regeneration in preclinical in vivo studies. Biology.

[15] McEwan J.K., Tribe H.C., Jacobs N., Hancock N., Qureshi A.A., Dunlop D.G., Oreffo R.O. (2018). Regenerative medicine in lower limb reconstruction. Regen. Ther..

[16] Einhorn T.A., Gerstenfeld L.C. (2015). Fracture healing: Mechanisms and interventions. Nat. Rev. Rheumatol..

[17] Williams D.F. (2008). On the mechanisms of biocompatibility. Biomaterials.

[18] Ikada Y. (2006). Challenges in tissue engineering. J. R. Soc. Interface.

[19] Howard D., Buttery L.D., Shakesheff K.M., Roberts S.J. (2008). Tissue engineering: Strategies, stem cells and scaffolds. J. Anat..

[20] Butscher A., Bohner M., Hofmann S., Gauckler L., Müller R. (2011). Structural and material approaches to bone tissue engineering in powder-based three-dimensional printing. Acta Biomater..

[21] Ma H., Feng C., Chang J., Wu C. (2018). 3D-printed bioceramic scaffolds: From bone tissue engineering to tumor therapy. Acta Biomater..

[22] Carotenuto F., Politi S., Haq A.U., De Matteis F., Tamburri E., Terranova M.L., Teodori L., Pasquo A., Di Nardo P. (2022). From Soft to Hard Biomimetic Materials: Tuning Micro/Nano-Architecture of Scaffolds for Tissue Regeneration. Micromachines.

[23] Chocholata P., Kulda V., Babuska V. (2019). Fabrication of scaffolds for bone-tissue regeneration. Materials.

[24] Yaszemski M.J., Trantolo D.J., Lewandrowski K.U., Hasirci V., Altobelli D.E., Wise D.L. (2003). Biomaterials in Orthopedics.

[25] Zhang D., Wu X., Chen J., Lin K. (2018). The development of collagen based composite scaffolds for bone regeneration. Bioact. Mater..

[26] Shi C., Yuan Z., Han F., Zhu C., Li B. (2018). Polymeric biomaterials for bone regeneration. Ann. Jt..

[27] Türk S., Altınsoy I., Çelebi Efe G., Ipek M., Özacar M., Bindal C. (2018). 3D Porous collagen/fulnctionalized multiwalled carbon nanotube/chitosan/hydroxiapatite composite scaffolds for bone tissue engineering. Mater. Sci. Eng. C.

[28] Zarif M. (2018). A review of chitosan-, alginate-, and gelatin-based biocomposites for bone tissue engineering. Biomaterials.

[29] Echave M.C., Saenz del Burgo L., Pedraz J.L., Orive G. (2017). Gelatin as Biomaterial for Tissue Engineering. Curr. Pharm. Des..

[30] Johari N., Madaah Hosseini H.R., Samadikuchaksaraei A. (2018). Novel fruoridates silk fibroin/TiO_2_ nanocomposite scaffolds for bone tissue engeneering. Mater. Sci. Eng. C.

[31] Zhu Z., Wang Y.M., Yang J., Luo X.S. (2017). Hyaluronic acid: A versatile biomaterial in tissue engineering. Plast. Aesthetic Res..

[32] Zhai P., Peng X., Li B., Liu Y., Sun H., Li X. (2019). The application of hyaluronic acid in bone regeneration. Int. J. Biol. Macromol..

[33] Klemm D., Heublein B., Fink H., Bohn A. (2005). Cellulose: Fascinating Biopolymer and Sustainable Raw Material. Angew. Chem. Int. Ed..

[34] Dwivedi R., Kumar S., Pandey R., Mahajan A., Nandana D., Katti D.S., Mehrotra D. (2020). Polycaprolactone as biomaterial for bone scaffolds: Review of literature. J. Oral Biol. Craniofacial Res..

[35] Gregor A., Filová E., Novák M., Kronek J., Chlup H., Buzgo M., Blahnová V., Lukášová V., Bartoš M., Nečas A. (2017). Designing of PLA scaffolds for bone tissue replacement fabricated by ordinary commercial 3D printer. J. Biol. Eng..

[36] Gentile P., Chiono V., Hatton P. (2014). An Overview of Poly (lactic-co-glycolic) Acid (PLGA)—Based Biomaterials for Bone Tissue Engineering. Int. J. Mol. Sci..

[37] Pan Z., Ding J. (2012). Poly (lactide-co-glycolide) porous scaffolds for tissue engineering and regenerative medicine. Interface Focus.

[38] Bakhtiarimoghadam B., Shirian S., Mirzaei E., Sharifi S., Karimi I., Gharati G., Takallu S., Nazari H. (2021). Comparison capacity of collagen hydrogel, mix-powder and in situ hydroxyapatite/collagen hydrogelscaffolds with and without mesenchymal stem cells and platelet-rich plasma in regeneration of critical sized bone defect in a rabbit animal model. J. Biomed. Mater. Res.-Part B Appl. Biomater..

[39] Lee D., Wufuer M., Kim I., Choi T.H., Kim B.J., Jung H.G., Jeon G., Lee G., Jeon O.H., Chang H. (2021). Sequential dual-drug delivery of BMP-2 and alendronate from hydroxyapatite-collagen scaffolds for enhanced bone regeneration. Sci. Rep..

[40] Tateno A., Asano M., Akita D., Toriumi T., Tsurumachi-Iwasaki N., Kazama T., Arai Y., Matsumoto T., Kano K., Honda M. (2019). Transplantation of dedifferentiated fat cells combined with a biodegradable type i collagen-recombinant peptide scaffold for critical-size bone defects in rats. J. Oral Sci..

[41] Vigni G.E., Cassata G., Caldarella G., Cirincione R., Licciardi M., Miceli G.C., Puleio R., D’Itri L., Lo Coco R., Camarda L. (2023). Improved Bone Regeneration Using Biodegradable Polybutylene Succinate Artificial Scaffold in a Rabbit Model. J. Funct. Biomater..

[42] He Y., Zhu T., Liu L., Shi X., Lin Z. (2018). Modifying collagen with alendronate sodium for bone regeneration applications. RSC Adv..

[43] Fu C., Jiang Y., Yang X., Wang Y., Ji W., Jia G. (2021). Mussel-inspired gold nanoparticle and plga/l-lysine-g-graphene oxide composite scaffolds for bone defect repair. Int. J. Nanomed..

[44] Huang K.H., Wang C.Y., Chen C.Y., Hsu T.T., Lin C.P. (2021). Incorporation of calcium sulfate dihydrate into a mesoporous calcium silicate/poly-ε-caprolactone scaffold to regulate the release of bone morphogenetic protein-2 and accelerate bone regeneration. Biomedicines.

[45] Lee S., Choi D., Shim J.H., Nam W. (2020). Efficacy of three-dimensionally printed polycaprolactone/beta tricalcium phosphate scaffold on mandibular reconstruction. Sci. Rep..

